# Cognitive Profile of Patients With Mitochondrial Chronic Progressive External Ophthalmoplegia

**DOI:** 10.3389/fneur.2020.00036

**Published:** 2020-01-29

**Authors:** Guanyu Zhang, Yue Hou, Zhaoxia Wang, Zheng Ye

**Affiliations:** ^1^Institute of Psychology, Chinese Academy of Sciences, Beijing, China; ^2^Department of Psychology, University of Chinese Academy of Sciences, Beijing, China; ^3^Department of Neurology, Peking University First Hospital, Beijing, China; ^4^Key Laboratory of Primate Neurobiology, Institute of Neuroscience, Center for Excellence in Brain Science and Intelligence Technology, Chinese Academy of Sciences, Shanghai, China

**Keywords:** mitochondrial chronic progressive external ophthalmoplegia, cognition, executive functions, language, working memory, memory, visuospatial functions

## Abstract

Mitochondrial chronic progressive external ophthalmoplegia (CPEO) is a major manifestation of human mitochondrial encephalomyopathies. Previous studies have shown cognitive deficits in patients with mitochondrial diseases. However, these studies often included patients with heterogeneous subtypes of mitochondrial diseases. Here, we aimed to provide a better cognitive profile of patients with CPEO by applying a comprehensive battery of neuropsychological assessments in a pure sample of patients with CPEO. We recruited 28 patients with CPEO (19 women, age 16–62 years) and 38 age- and education-matched healthy control subjects (25 women, age 16–60 years). The neuropsychological assessments covered global cognition and five cognitive domains (executive functions, language, working memory, memory, and visuospatial functions). We found that the patients were impaired in global cognition [Montreal Cognitive Assessment (MoCA)], executive functions [Trail Making Test Part B (TMT-B)], and language [Boston Naming Test (BNT)], but not in working memory, memory or visuospatial functions. Moreover, individual patients' performances in the TMT-B (completion time) were predicted by the severity of non-ophthalmoplegia mitochondrial symptoms/signs [Newcastle Mitochondrial Disease Adult Scale (NMDAS)] and duration of the mitochondrial disease (years). Namely, patients with more severe non-ophthalmoplegia mitochondrial symptoms/signs and a longer disease duration took a longer time to complete the TMT-B. No clinical measures predicted individual patients' performances in the BNT.

## Introduction

Mitochondrial encephalomyopathies are a heterogeneous group of inherited multisystem disorders that predominantly affect tissues with major aerobic metabolisms, including the central nervous system, muscle, retina, and tubular epithelium (1, 2). The major manifestations of mitochondrial encephalomyopathy include mitochondrial encephalopathy, lactic acidosis and stroke-like episodes (MELAS), chronic progressive external ophthalmoplegia (CPEO), Kearn-Sayre syndrome (KSS), and myoclonus epilepsy with ragged-red fibers syndrome (MERRF). In this study, we aimed to provide a cognitive profile of patients with CPEO by assessing their global cognition and five cognitive domains (executive functions, language, working memory, memory, and visuospatial functions).

Previous studies have systematically examined cognitive deficits and brain structural alterations in patients with MELAS. Symptoms of MELAS include epileptic seizures, transient ischemic attack, lactic acidosis, and ragged red fibers in muscle biopsy. MELAS has been associated with various point mutations. The most common mutation is m.3243A>G mutation (3). Neuropsychological studies showed that patients with MELAS are impaired in general intelligence (Wechsler Intelligence Scale), executive functions (Wisconsin Card Sorting Test and Trail Making Test), language [Boston Naming Test (BNT) and Verbal Fluency Test], attention/working memory (Digit Span Test), memory (Auditory Verbal Learning Test), and visuospatial functions (Rey-Osterrieth Complex Figure Test and Block Design Test) (4–6). Moreover, patients with MELAS often show more severe deficits in working memory, memory, and visuospatial functions than patients with CPEO and patients with KSS (7). Brain imaging studies showed that patients with MELAS have a higher water and lactate level in the parietal and occipital regions, as well as calcification of the basal ganglia (6, 8).

The cognitive impact of CPEO is less understood. Symptoms of CPEO include progressive weakness of extraocular muscles, with or without extra-ocular manifestations. CPEO includes both sporadic and autosomal dominant and recessive inheritance. Sporadic CPEO is associated with single large-scale mitochondrial DNA deletions or point mutations. Autosomal dominant and recessive CPEO is associated with nuclear gene mutations which often cause secondary multiple mitochondrial DNA deletions (9). Because CPEO is rare (the incidence is only 1–2 in 100,000), previous studies on cognitive impairment often included a heterogeneous group of patients with CPEO and patients with other subtypes of mitochondrial diseases in order to reach an appropriate sample size. For example, Turconi et al. (10) included patients with CPEO, patients with KSS, and patients with MERRF. Bosbach et al. (11) included patients with CPEO and patients with KSS. These studies reported deficits in executive functions (Wisconsin Card Sorting Test), working memory (Digit Span Test), and visuospatial functions (Block Design Test and Rey Figure Copy Test), but not in general intelligence. It is unclear whether patients with CPEO are impaired in all three specific domains or only some of them.

To provide a better cognitive profile of patients with CPEO, we measured global cognition and five cognitive domains in a pure sample of patients with CPEO. We used the Montreal Cognitive Assessment (MoCA) and the Wechsler Adult Intelligence Scale (WAIS) to evaluate global cognition; the Trail Making Test Part B (TMT-B) for executive functions, the BNT and Animal Fluency Test for language, the Digit Span Forward Test and Adaptive Digit Ordering Test for working memory, the Rey's Auditory Verbal Learning Test for memory, and the Block Design Test and Clock Drawing Test for visuospatial functions (11–14). These tests were selected because (1) a revised and validated version of each test was readily available in China; and (2) the tests were less visually demanding (e.g., compared to the Rey-Osterrieth Complex Figure Test). We first examined whether the patients with CPEO were impaired in global cognition. After observing the global cognitive impairment, we then measured cognitive functions in each specific domain. Finally, we examined whether domain-specific cognitive impairment can be predicted by clinical features.

## Materials and Methods

This study was approved by the ethics committee of Peking University First Hospital in accordance with the Declaration of Helsinki. Each participant signed a written informed consent before participating in the study. For participants under the age of 18 years, their legal guardians signed the written informed consent.

### Patients and Clinical Assessments

We recruited 28 patients with CPEO at the Peking University First Hospital Department of Neurology between September 2017 and October 2018. Inclusion criteria were (1) diagnosed with CPEO [modified diagnostic criteria for mitochondrial respiratory chain disorders (15)]; (2) age 16–65 years; (3) having completed primary education (≥5 years); (4) Mandarin Chinese speaking. Exclusion criteria were (1) symptoms of other mitochondrial diseases (e.g., stroke-like episode); (2) a history of other major neurologic (e.g., epilepsy) or psychiatric diseases (e.g., schizophrenia), stroke or brain injury; (3) alcohol or substance abuse; (4) severe ptosis, or visual or hearing deficits that may impair performance in cognitive assessments.

All patients were diagnosed by an experienced neurologist (Z.W.). Table 1 presents the demographic features, clinical phenotypes, brain structures, genotypes, and drugs of individual patients. All patients showed external ophthalmoplegia. Other clinical phenotypes included exercise intolerance (10 patients), muscle weakness (six patients), dysarthria (six patients), dysphagia (five patients), dyspnea (two patients), peripheral neuropathy (two patients), hearing loss (two patients), migraine (one patient), diabetes mellitus (one patient), and premature ovarian failure (one patient). We evaluated the severity of the mitochondrial disease using the Newcastle Mitochondrial Disease Adult Scale (NMDAS) (16). To assess the severity of ophthalmoplegia and that of other mitochondrial symptoms/signs, we separately calculated the ophthalmoplegia relevant items (items 2 and 3 of the current clinical assessment section) and the non-ophthalmoplegia items (all other items).

**Table 1:** Demographic features, clinical phenotypes, brain structures, genotypes, and drugs of individual patients.

**Patients**	**Sex**	**Age (years)**	**Education (years)**	**Clinical phenotypes**	**Brain MRI**	**Genotypes**	**Drugs (mg/day)**
1	F	32	8	EO, EI	Normal	SLD	Vitamin B2 (15), idebenone (90)
2	M	62	10	EO, EI, MW, DAR, PN, DM	Normal	SLD	Idebenone (90), alpha-lipoic acid (300), creatine monohydrate (5,000), metformin (1,000)
3	M	58	16	EO, EI, MW, DAR	Normal	SLD	Levocarnitine (3,000), idebenone (90), creatine monohydrate (5,000)
4	F	27	19	EO	Normal	SLD	Vitamin C (100), vitamin E (200), coenzyme Q10 (30)
5	M	47	13	EO	Normal	SLD	Vitamin B2 (15), idebenone (90)
6	F	21	14	EO	Normal	SLD	None
7	F	21	16	EO, DPH	Normal	SLD	Levocarnitine (3,000), idebenone (90)
8	F	32	15	EO	Normal	SLD	Vitamin B1 (30), vitamin B2 (15), coenzyme Q10 (100)
9	F	22	17	EO	Normal	SLD	None
10	F	38	5	EO	Normal	SLD	None
11	M	20	12	EO, EI, DAR	Normal	SLD	Levocarnitine (3,000), coenzyme Q10 (100)
12	F	27	15	EO	Normal	SLD	Vitamin E (200), coenzyme Q10 (30)
13	F	34	18	EO, PN, POF	Normal	*POLG*	Levocarnitine (3,000), coenzyme Q10 (100), alpha-lipoic acid (300)
14	F	36	12	EO	Normal	SLD	None
15	F	44	15	EO, EI, MW, DAR, DPH	Normal	SLD	Vitamin B2 (15), levocarnitine (3,000), idebenone (90), creatine monohydrate (5,000)
16	M	37	22	EO, EI	Normal	SLD	None
17	F	34	19	EO	Normal	SLD	Vitamin B2 (15), vitamin C (100), vitamin E (200)
18	M	36	17	EO, EI, MW, DA, DPH	Normal	SLD	Levocarnitine (3,000), coenzyme Q10 (300), creatine monohydrate (5,000)
19	F	26	9	EO	Normal	SLD	None
20	M	22	15	EO	Normal	SLD	None
21	M	29	18	EO	Normal	SLD	None
22	M	39	9	EO	Normal	SLD	None
23	F	16	10	EO	Normal	SLD	None
24	F	43	9	EO, EI, MW, DAR, DPH, DPN, HL, MI	High T2 signals in subcortical white matter	*RRM2B*	Vitamin B2 (15), levocarnitine (3,000), coenzyme Q10 (300), creatine monohydrate (5,000)
25	F	25	16	EO, EI	Normal	SLD	None
26	F	42	16	EO	Normal	*TK2*	Idebenone (90), creatine monohydrate (5,000)
27	F	46	15	EO	Normal	SLD	None
28	F	38	9	EO, EI, MW, DAR, DPH, DPN, HL	High T2 signals in subcortical white matter	*RRM2B*	Vitamin B2 (15), levocarnitine (3,000), coenzyme Q10 (300), creatine monohydrate (5,000)

*MRI, magnetic resonance imaging; F, female; M, male; EO, external ophthalmoplegia; EI, exercise intolerance; MW, muscle weakness; DAR, dysarthria; DPH, dysphagia; DPN, dyspnea; PN, peripheral neuropathy; HL, hearing loss; MI, migraine; DM, diabetes mellitus; POF, premature ovarian failure; SLD, single large-scale mitochondrial DNA deletion; POLG, DNA polymerase gamma; RRM2B, ribonucleotide reductase M2B (TP 53 inducible); TK2, thymidine kinase 2*.

All patients completed a brain magnetic resonance imaging examination. Two of the patients showed symmetric high T2 signals in cerebral subcortical white matter, indicating that they had leukoencephalopathy.

The clinical diagnosis was confirmed by muscle histopathology and genetic examinations (17). All patients showed ragged red or blue fibers, and cytochrome oxidase deficiency fibers on muscle biopsies (18). The genetic test was performed following a standard protocol as previously reported (19–21). Twenty-four sporadic patients had single large-scale mitochondrial DNA deletions and four autosomal inherited patients had nuclear gene mutations, including ribonucleotide reductase M2B (two patients), DNA polymerase gamma (one patient), and thymidine kinase 2 (one patient).

All patients were assessed under their regular treatment, including vitamins (nine patients), coenzyme Q10 (eight patients), levocarnitine (eight patients), idebenone (seven patients), creatine monohydrate (seven patients), alpha-lipoic acid (two patients), or metformin (one patient).

### Neuropsychological Assessments

We first evaluated the global cognition with the MoCA (22) and the Wechsler Adult Intelligence Scale-Revised in China (WAIS-RC) (23, 24). Note, the WAIS-RC was designed for individuals older than 16 years. We then assessed the patients' performances in five cognitive domains including executive functions (TMT-B), language (BNT and Animal Fluency Test), working memory (Digit Span Forward Test and Adaptive Digit Ordering Test), memory (Rey's Auditory Verbal Learning Test), and visuospatial functions (Clock Drawing Test and Block Design Test). Below we briefly describe each test and its key parameters.

#### Executive Functions

In the TMT-B (25), participants were asked to connect a sequence of 25 numbers in the ascending order as quickly as possible, and to alternate between numbers in squares and numbers in circles (e.g., 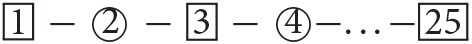). Key parameters of the test included the time used to complete the test and the number of errors.

#### Language

In the BNT (14, 26, 27), participants were given a set of 30 pictures and asked to name each picture within 20 s. They would be given a semantic or phonemic cue if they failed to name a particular picture correctly. We scored the total number of correct responses with or without semantic cues. In the Animal Fluency Test (28), participants were asked to name as many animals as possible in 1 min. We scored the total number of correct responses by excluding duplicates and by excluding category names when specific examples were given (e.g., excluding “fish,” when it was followed by “goldfish”).

#### Working Memory

In the Digit Span Forward Test (24, 29, 30), participants heard a sequence of 3–12 random digits at the rate of one digit per s in each trial. They were asked to immediately recall the digits in the original order. The test was adaptive regarding the length of each trial and was terminated when participants failed in both trials of a particular length. The Adaptive Digit Ordering Test (31) was similar to the digit span forward test except that participants were asked to recall the digits in ascending order. For each test, we scored the length of the last correctly recalled trial (span).

#### Memory

In Rey's Auditory Verbal Learning Test (14, 32), participants were asked to learn 15 words and to recall as many words as possible five times immediately, and once after 30 min. We scored the total number of correctly recalled words in the immediate recalls and the number of correctly recalled words in the delayed recall.

#### Visuospatial Functions

In the Clock Drawing Test (14, 33), participants were asked to draw a clock, to place the numbers in the correct positions, and to draw the hands to indicate 10 past 11. We used the scoring method Shulman (34). In the Block Design Test (14, 24), participants were asked to arrange a set of nine blocks to match a given pattern as quickly as possible. We used the scoring method of Peng and Zhang (14).

We additionally monitored mood and sleep symptoms using the Beck Depression Inventory-II (BDI-II) (35) and REM Sleep Behavior Disorder Screening Questionnaire (RBDSQ) (36).

### Healthy Control Subjects

We recruited 38 age- and education-matched healthy control subjects. Exclusion criteria were (1) a history of significant neurologic or psychiatric disorders, stroke, or brain injury; (2) possible dementia or mild cognitive impairment (MoCA ≥ 26/30); (3) possible current depression (BDI-II ≤ 7); (4) alcohol abuse or use of addictive drugs (e.g., opioid drugs). They completed the same assessments for cognition, mood, and sleep as the patients.

### Statistical Analysis

Data were processed with IBM SPSS Statistics 20. First, we examined whether the patients were different from the healthy control subjects in global cognition (MoCA and WAIS-RC) using two-sample *t*-tests (two-tailed, *p* < 0.025 Bonferroni correction for two tests). Given the presence of deficits in global cognition, we then examined whether the patients performed worse than healthy control subjects in each cognitive domain using two-sample *t*-tests (one-tailed, *p* < 0.005 Bonferroni correction for 10 tests). After observing group differences in executive functions and language, we finally used linear regression models to examine whether individual patients' performances in the TMT-B (the completion time) or BNT (the number of correct responses) can be statistically predicted by their clinical features (*p* < 0.025 Bonferroni correction for two models). Independent variables were the severity of ophthalmoplegia (ophthalmoplegia relevant NMDAS subscore), the severity of other mitochondrial symptoms/signs (non-ophthalmoplegia NMDAS subscore), disease duration (years), weight status (body mass index), and sleep symptoms (RBDSQ score). The body mass index and the RBDSQ score were included because they showed significant group differences (see Table 2).

**Table 2:** Demographic and clinical features of the patients and healthy control subjects (means, standard deviations, and group differences).

**Features/measures**	**Patients (*N* = 28)**	**Healthy controls (*N* = 38)**	**Group differences (*p* values)**
Male:Female	9:19	13:25	0.860
Age (years)	34.1 (11.2)	36.6 (12.6)	0.410
Education (years)	13.9 (4.1)	13.6 (2.8)	0.765
Duration of disease (years)	13.4 (10.9)	–	–
Newcastle mitochondrial disease adult scale	8.9 (6.9)	–	–
Ophthalmoplegia relevant	6.3 (3.0)	–	–
Other mitochondrial symptoms/signs	2.6 (4.7)	–	–
Body mass index	19.9 (3.1)	23.1 (3.2)	0.001^*^
Beck depression inventory II	5.6 (6.1)	2.4 (2.1)	0.012
REM sleep behavior disorder screening questionnaire	3.8 (2.4)	2.2 (1.5)	0.002^*^

Group differences, p values of two-sample t-tests, or Chi-square test as appropriate; asterisks (^*^), a significant difference (p < 0.008 Bonferroni correction for six tests).

## Results

### Demographic and Clinical Features

Table 2 shows the demographic and clinical features of the patients and healthy control subjects. The two-sample *t*-tests (two-tailed, *p* < 0.008 Bonferroni correction for six tests) revealed significant group differences in the body mass index [*t*_(64)_ = 4.14, *p* < 0.001] and RBDSQ score [*t*_(64)_ = 3.23, *p* = 0.002]. The patients had a lower body mass index and a higher RBDSQ score. The underweight of the patients may be due to the dysfunction of aerobic energy production (2). The potential RBD sleep problems are not often reported in patients with mitochondrial disorders but are common in neurodegenerative disorders such as Parkinson's disease.

### Global Cognition

Table 3 shows measures of the patients and healthy control subjects in global cognition and in each cognitive domain. The two-sample *t*-tests (two-tailed, *p* < 0.025 Bonferroni correction for two tests) revealed a significant group difference in the MoCA [*t*_(64)_ = 2.34, *p* = 0.025] but not in the WAIS-RC [*t*_(64)_ = 1.69, *p* = 0.099]. MoCA may be more specific to the types of cognitive deficits in patients with CPEO.

**Table 3:** Cognitive measures of the patients and healthy control subjects (means, standard deviations, and group differences).

**Measures**	**Patients (*N* = 28)**	**Healthy controls (*N* = 38)**	**Group differences (*p* values)**
**Global cognition**			
Montreal cognitive assessment	26.1 (3.0)	27.5 (1.4)	0.025^*^
Wechsler adult intelligence scale-revised in China	105.7 (18.1)	112.3 (11.4)	0.099
**Executive functions**			
Trail making test part B: completion time	143.7 (71.1)	104.8 (31.8)	0.006
Trail making test part B: total errors	0.1 (0.4)	0.6 (0.8)	0.005^*^
**Language**			
Boston naming test: correct responses	22.6 (3.4)	24.9 (2.8)	0.003^*^
Animal fluency test: correct responses	20.0 (5.4)	21.6 (4.3)	0.093
**Working memory**			
Digit span forward test: span	8.3 (1.3)	8.4 (0.8)	0.397
Adaptive digit ordering test: span	6.0 (1.4)	6.5 (1.1)	0.054
**Memory**			
Rey's auditory verbal learning test: immediate recalls	49.4 (10.5)	51.6 (9.3)	0.195
Rey's auditory verbal learning test: delayed recall	10.5 (3.2)	10.8 (2.3)	0.333
**Visuospatial functions**			
Clock drawing test (max. 5 points)	4.7 (0.7)	4.7 (0.6)	0.342
Block design test (max. 55 points)	30.7 (11.4)	34.1 (8.0)	0.081

Group differences, p values of two-sample t-tests; asterisks (^*^), a significant difference (global cognition: p < 0.025 Bonferroni correction for two tests; five cognitive domains: p < 0.005 Bonferroni correction for 10 tests).

### Five Cognitive Domains

Having observed the deficits in global cognition, we then examined whether the patients performed worse than healthy control subjects in each cognitive domain using two-sample *t*-tests (one-tailed, *p* < 0.005 Bonferroni correction for 10 tests). In brief, the patients were impaired in executive functions and language but not in working memory, memory or visuospatial functions (see Table 3).

For executive functions, the patients tended to perform more slowly [*t*_(63)_ = 2.66, *p* = 0.006] but not to make more errors than healthy control subjects in the TMT-B. In the language domain, the patients scored lower than healthy control subjects in the BNT [*t*_(61)_ = 2.91, *p* = 0.003], but not in the Animal Fluency Test [*t*_(64)_ = 1.34, *p* = 0.093]. We obtained no group difference in working memory, memory, or visuospatial functions.

### Relationship Between Executive Dysfunction and Clinical Features

**Figure 1:**
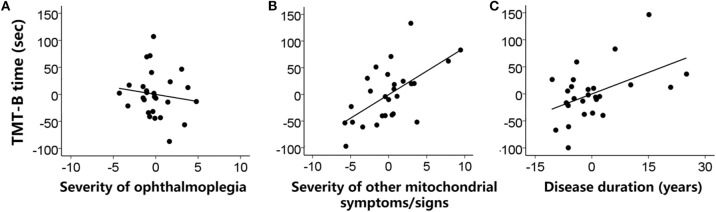
Individual patients' performances in the TMT-B (completion time) were predicted **(A)** not by the severity of ophthalmoplegia (ophthalmoplegia relevant NMDAS subscore) but by **(B)** the severity of other mitochondrial symptoms/signs (non-ophthalmoplegia NMDAS subscore) and **(C)** the disease duration (years) when weight status and sleep symptoms were controlled. Values were demeaned.

Figure 1 shows the relationship between the measures of executive functions and clinical features. The linear regression model for the TMT-B was significant [*F*_(5,21)_ = 8.02, *p* < 0.001, *R*^2^ = 0.57]. The TMT-B completion time was statistically predicted by the severity of non-ophthalmoplegia mitochondrial symptoms/signs (beta = 8.84, *t* = 3.52, *p* = 0.002) and the disease duration (beta = 2.66, *t* = 2.56, *p* = 0.018), but not by the severity of ophthalmoplegia (beta = −2.64, *t* < 1). The patients with more severe non-ophthalmoplegia mitochondrial symptoms/signs and a longer disease duration performed more slowly in the TMT-B.

### No Relationship Between Language and Clinical Features

The linear regression model for the BNT was not significant [*F*_(5,21)_ = 1.17, *p* = 0.356, *R*^2^ = 0.03]. The patients' performances in the BNT were not predicted by the severity of ophthalmoplegia, the severity of non-ophthalmoplegia mitochondrial symptoms/signs, or other clinical features.

## Discussion

In this study, we measured the global cognition and five cognitive domains in patients with CPEO and healthy control subjects. The patients were impaired in global cognition and in executive functions and language, but not in working memory, memory or visuospatial functions. To be specific, the patients scored lower in MoCA. They tended to perform more slowly in the TMT-B and to name fewer pictures in the BNT. Individual patients' performances in the TMT-B, but not in the BNT, were predicted by the severity of non-ophthalmoplegia mitochondrial symptoms/signs and the duration of the mitochondrial disease. Patients who had more severe non-ophthalmoplegia mitochondrial symptoms/signs and a longer disease duration tended to perform more slowly in the TMT-B.

This study is consistent with previous findings that patients with CPEO performed equally well as healthy control subjects in the WAIS (11). However, we observed a worse MoCA in patients with CPEO, which suggests the existence of the deterioration in global cognition in these patients. The type of assessment made by MoCA may be more specific to the types of deficits in patients with CPEO (e.g., executive dysfunction).

We also confirmed that patients with CPEO are impaired in executive functions. Previous studies showed that a heterogeneous group of patients with CPEO and patients with KSS made more preservative errors than healthy control subjects in the Wisconsin Card Sorting Test (11). Here we demonstrated that patients with CPEO took a longer time to complete the TMT-B than healthy control subjects. The Wisconsin Card Sorting Test and the TMT-B share a common feature that participants have to shift frequently between rules or items. Together, these findings suggest that patients with CPEO may be impaired in task switching and set-shifting. A novel finding of this study is that individual patients' differences in the TMT-B were predicted by the severity of non-ophthalmoplegia mitochondrial symptoms/signs. It suggests that the patients' poor performances in the TMT-B was unlikely due to saccade problems (e.g., moving visual fixation from one number or letter to the next).

Regarding language deficits, previous studies showed that patients with CPEO or KSS were impaired in word comprehension (11). This study suggests that patients with CPEO may also be impaired in object naming, even though they seemed to perform normally in animal name generation. Similar deficits in object naming have been reported in patients with MELAS (37). As patients with MELAS do not often have significant ophthalmoparesis, the underlying pathophysiology of the naming deficits is likely beyond the ophthalmoplegia. This hypothesis is supported by the absence of a correlation between the naming performance and the severity of ophthalmoplegia in this study.

In mitochondrial diseases, mutant mitochondrial DNA accumulates in the frontal lobe more than the parietal, occipital, or temporal lobes (38). The progressive accumulation of mutant mitochondrial DNA in the frontal lobe may lead to increased oxidative metabolism stress that impairs functions of frontal neurons and glial cells (39, 40) and consequently may harm the cognitive functions that heavily rely on the frontal lobe (e.g., executive functions and language).

We did not find deficits in working memory, memory, or visuospatial functions, which have been reported by previous studies that included patients with heterogeneous subtypes of mitochondrial diseases. The inconsistency may come from the variability in working memory deficits, memory deficits, and visuospatial deficits in the subtypes of mitochondrial diseases. For example, Lang et al. (7) showed that patients with MELAS tended to perform worse than patients with CPEO in working memory, memory, and visuospatial tests. Bosbach et al. (11) showed that patients with KSS scored abnormally lower in 31% of the neuropsychological tests, while patients with CPEO only scored abnormally in 24% of the neuropsychological tests (11). Potential deficits in working memory, memory, or visuospatial functions may not be central to the cognitive profile of patients with CPEO.

In conclusion, we provided a cognitive profile of patients with CPEO. Patients with CPEO were impaired in global cognition and specifically in executive functions and language. Individual patients' differences in executive functions can be predicted by the severity of non-ophthalmoplegia mitochondrial symptoms/signs and the duration of the mitochondrial disease. CPEO patients with more severe non-ophthalmoplegia mitochondrial symptoms/signs and a longer disease duration tended to be worse in executive functions. Executive dysfunction may be a characteristic cognitive feature of patients with CPEO. Future research needs to explore the neural circuits underlying the executive dysfunction (e.g., impaired set-shifting) and potential therapeutic approaches for enhancing executive functions in patients with CPEO.

## Data Availability Statement

The datasets generated for this study are available on request to the corresponding author.

## Ethics Statement

This study involving human participants was reviewed and approved by the ethics committee of Peking University First Hospital. Written informed consent to participate in this study was provided by the participants or their legal guardian/next of kin.

## Author Contributions

ZY and ZW designed and organized the study. GZ and YH collected the data. GZ analyzed the data. GZ and ZY wrote the first draft of the manuscript. ZW and YH revised the manuscript. All authors approved the submitted version.

### Conflict of Interest

The authors declare that the research was conducted in the absence of any commercial or financial relationships that could be construed as a potential conflict of interest.
